# Isolated Submandibular Sialadenitis in a Preterm Neonate: A Rare Cause of Neck Swelling

**DOI:** 10.7759/cureus.98054

**Published:** 2025-11-28

**Authors:** Maria Shahid, Aaya Seedahmed, Shehan Liyanage

**Affiliations:** 1 Neonatology, Royal Stoke University Hospital, University Hospitals of North Midlands NHS Trust, Stoke-on-Trent, GBR

**Keywords:** cervical swelling, neonatal infection, neonatal sialadenitis, preterm neonate, salivary gland inflammation, submandibular gland

## Abstract

Sialadenitis is an infection or inflammation of the salivary glands and can be caused by bacteria, viruses, or obstruction, with *Staphylococcus aureus *being the primary cause*.* Risk factors for neonatal sialadenitis include prematurity, immunological immaturity, dehydration, tube feeding, and factors predisposing to bacterial colonization. Symptoms may manifest as localized swelling, systemic signs of infection, or respiratory instability in preterm neonates. Isolated submandibular sialadenitis is extremely rare, with only a few cases reported in the medical literature. We report a case of a preterm neonate with isolated submandibular sialadenitis, in which culture and swabs were negative, but ultrasound findings suggested infection of the submandibular gland. Ultrasound played a key role in diagnosis, as serial scans in this case confirmed sialadenitis and guided treatment. The patient received intravenous flucloxacillin, gentamicin, and metronidazole, resulting in complete resolution of the swelling. Early treatment is crucial to prevent serious complications. Awareness of this rare condition can prevent delayed diagnosis and reduce the risk of serious complications.

## Introduction

Sialadenitis is an inflammation or infection of the salivary glands, which may be acute, chronic, or recurrent. It is most commonly caused by bacterial or viral infections, with *Staphylococcus aureus* being the primary cause, and may also result from ductal obstruction [1]. The parotid gland is most frequently affected as it is the largest salivary gland and produces serous secretions that are considered less immunogenic [1]. Sialadenitis is highly uncommon in neonates, typically presenting as acute suppurative parotitis or suppurative submandibular sialadenitis [2]. Risk factors associated with neonatal sialadenitis include prematurity, immunological immaturity, low birth weight, dehydration, and nasogastric tube feeding [3]. Clinical manifestations may include localized swelling, systemic signs of infection, or respiratory instability in preterm neonates. Isolated submandibular sialadenitis is extremely rare, with only a few cases reported in the literature [4]. *S. aureus* remains the predominant pathogen in neonatal submandibular sialadenitis, with *Pseudomonas aeruginosa*, *Klebsiella pneumoniae*, and *Enterococcus faecalis* also reported, supporting the need for broad-spectrum antibiotics for treatment [3]. Because of its low incidence, precise prevalence data for neonatal submandibular sialadenitis remain unclear, and the condition may be misdiagnosed or overlooked. Awareness is essential, as untreated sialadenitis can lead to serious complications, including abscess formation, systemic infection, and airway compromise [5]. Early recognition and appropriate antibiotic therapy generally yield favourable outcomes.

We report a case of submandibular sialadenitis in a preterm neonate born at 30+1 weeks of gestation. This case highlights the diagnostic challenges and emphasizes the importance of early recognition and management to avoid adverse outcomes.

## Case presentation

**​​​**A 1300-gram male dichorionic diamniotic (DCDA) twin was delivered at 30+1 weeks’ gestation via emergency cesarean section due to intrauterine growth restriction (IUGR) and absent end-diastolic flow on Doppler ultrasound. Antenatal history was unremarkable. The neonate was in good condition at birth, required minimal respiratory support with continuous positive airway pressure (CPAP) and high-flow nasal oxygen, and remained clinically stable. A sepsis screen was performed on admission, and due to neutropenia, a five-day course of antibiotics was administered. The infant remained well with no subsequent concerns.

On day 14 of life, nursing staff noted a firm 2 × 2.5 cm swelling in the left submandibular region. The infant experienced intermittent episodes of desaturation, bradycardia, and apnea, one of which required positive-pressure face-mask ventilation; overall clinical observations remained stable. Bedside ultrasound demonstrated enlargement of the left submandibular gland, but was otherwise inconclusive due to ultrasound equipment limitations. A repeat ultrasound was planned once the infant became clinically stable.

Given the swelling and cardiorespiratory instability, a C-reactive protein (CRP) test was performed, which was elevated, prompting a full sepsis screen, and the infant was started on IV antibiotics. A repeat ultrasound suggested submandibular sialadenitis. Following consultation from the ENT team, antibiotic therapy was extended to complete a 10-day course.

Investigations

Sepsis screen revealed a white blood cell (WBC) count of 18.16 × 10⁹/L (reference range 7.3-16.6 × 10⁹/L), with CRP trending from 44 mg/L (reference range 0-5 mg/L) initially, to 23 mg/L, 10 mg/L, and <4 mg/L over 10 days. Blood cultures were negative. Serology and swabs for Epstein-Barr virus (EBV) and cytomegalovirus (CMV) were also negative.

**Figure 1:**
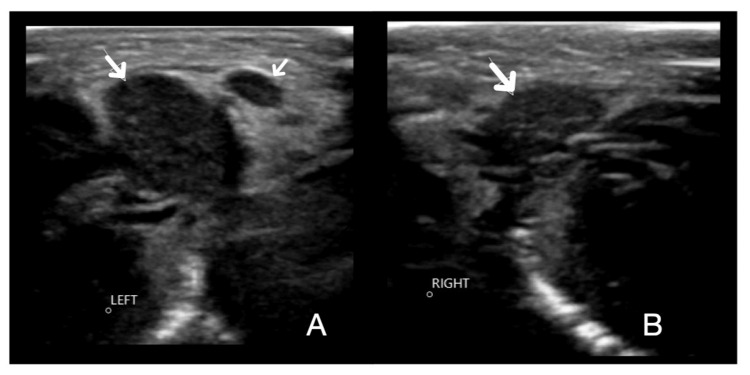
Ultrasound scan of the left and right submandibular glands. (A) Left submandibular gland showing diffuse enlargement (arrow), increased vascularity, and a small local lymph node (arrow) suggestive of inflammation. (B) Right submandibular gland for comparison (arrow).

Initial neck ultrasound on day 15 of life demonstrated diffuse enlargement of the left submandibular gland (Figure 1A) relative to the right (Figure 1B), with increased vascularity and echogenicity of the surrounding subcutaneous tissues and a small local lymph node (Figure 1A) suggestive of inflammation. The gland itself appeared homogeneous and symmetrical. This ultrasound scan was inconclusive due to the frequency and size of the portable linear probe.

**Figure 2:**
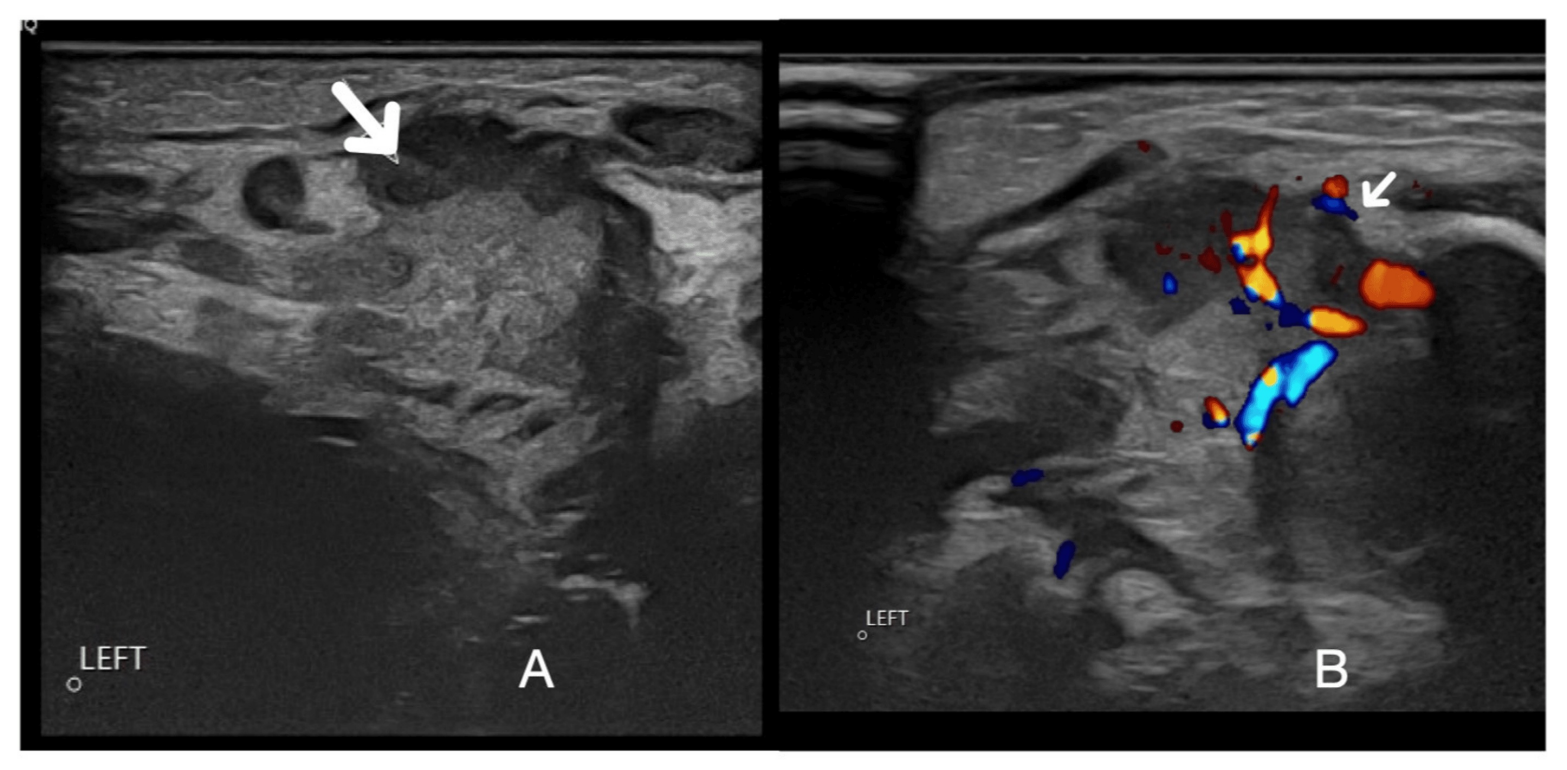
Repeat ultrasound scan of the left submandibular gland. (A) Left submandibular gland showing diffuse enlargement and heterogeneity (arrow). (B) Hyperaemia of the gland (arrow)  and continued surrounding soft-tissue inflammation.

Repeat ultrasound with a high-frequency linear probe on day 23 of life revealed diffuse enlargement and heterogeneity of the left submandibular gland (Figure 2A) with hyperaemia of the gland (Figure 2B) and continued surrounding soft-tissue inflammation, consistent with acute sialadenitis. There was no focal lesion or liquefied component. No dilated duct or stone was seen.

Differential diagnosis

Cervical swelling in preterm neonates can result from a variety of congenital, infectious, or inflammatory conditions. Congenital swellings include branchial cleft cysts, thyroglossal duct cysts, lymphatic malformations such as cystic hygroma, and rare congenital tumors such as sternocleidomastoid tumor. Infective and inflammatory causes, including neonatal sialadenitis and reactive lymphadenopathy, should also be considered. Given the firm swelling and raised inflammatory markers, an infective etiology was considered most likely. Ultrasound imaging subsequently supported a diagnosis of submandibular sialadenitis.

Treatment

The patient initially received a five-day course of intravenous flucloxacillin and gentamicin, along with metronidazole, based on microbiology advice. After the repeat ultrasound and ENT consultation, antibiotic therapy was continued for a total of 10 days.

Outcome and follow-up

The patient was discharged in stable condition. Follow-up with pediatric ENT confirmed complete resolution of the swelling.

## Discussion

This case report describes a preterm neonate with isolated submandibular sialadenitis, which was culture and swab negative; however, ultrasound findings showed inflammation of the submandibular gland. Isolated infection of the submandibular gland is infrequent [4]. The rarity of submandibular sialadenitis in this age group, coupled with its subtle presentation, highlights its diagnostic importance. In this patient, swelling developed at two weeks of life, accompanied by signs of systemic infection. The swelling resolved completely after a 10-day course of antibiotics.

In preterm babies, risk factors include prematurity, prolonged nasogastric feeding, and dehydration [6]. In our patient, no ductal obstruction or stone was identified, suggesting that the infection likely originated from bacteria ascending naturally. Investigations should include blood tests, inflammatory markers, blood cultures, and imaging. Ultrasound is the preferred diagnostic modality, as it shows gland size, texture, and surrounding tissue inflammation without radiation [7]. Serial ultrasound scans in our case confirmed sialadenitis and guided treatment. Diagnosis can be difficult because neck swelling in neonates has a broad differential, including congenital cysts, lymphatic malformations, and reactive lymphadenopathy. 

Antibiotic therapy is the mainstay of treatment. Given the range of potential pathogens in neonatal submandibular sialadenitis, early initiation of broad-spectrum intravenous antibiotics is recommended while awaiting culture results. In this case, the patient responded well to intravenous flucloxacillin, gentamicin, and metronidazole. Surgery or drainage is rarely needed unless an abscess forms [3]. Early treatment prevents serious complications, such as airway compromise or worsening infection [5]. This case highlights the need to consider submandibular sialadenitis in preterm neonates presenting with neck swelling, even when cultures are negative. In addition, multidisciplinary input supported timely diagnosis and management.

## Conclusions

Submandibular sialadenitis in preterm neonates is not only rare but also presents with its diagnostic challenges. The importance of prompt diagnosis and appropriate antibiotic therapy is demonstrated in preventing adverse outcomes. Ultrasound plays a central role in confirming diagnosis and guiding treatment, and the case shows the value of coordinated multidisciplinary care. Clinicians should consider submandibular sialadenitis in any preterm infant presenting with neck swelling, even when blood cultures are negative, as timely diagnosis and treatment can lead to excellent outcomes.
